# Insights into the Development of Phototrophic Biofilms in a Bioreactor by a Combination of X-ray Microtomography and Optical Coherence Tomography

**DOI:** 10.3390/microorganisms9081743

**Published:** 2021-08-16

**Authors:** Susanne Schaefer, Jakob Walther, Dorina Strieth, Roland Ulber, Ulrich Bröckel

**Affiliations:** 1 Institute of Microprocess Engineering and Particle Technology, University of Applied Sciences Trier, Environmental Campus Birkenfeld, P.O. Box 1380, 55761 Birkenfeld, Germany; s.schaefer@umwelt-campus.de (S.S.); u.broeckel@umwelt-campus.de (U.B.); 2 Institute of Bioprocess Engineering, University of Kaiserslautern, Gottlieb-Daimler-Str. 49, 67663 Kaiserslautern, Germany; walther@mv.uni-kl.de (J.W.); strieth@mv.uni-kl.de (D.S.)

**Keywords:** biofilm, optical coherence tomography, X-ray microtomography, biocarrier, cyanobacteria, MBBR, OCT, µCT, carrier, photobioreactor

## Abstract

As productive biofilms are increasingly gaining interest in research, the quantitative monitoring of biofilm formation on- or offline for the process remains a challenge. Optical coherence tomography (OCT) is a fast and often used method for scanning biofilms, but it has difficulty scanning through more dense optical materials. X-ray microtomography (μCT) can measure biofilms in most geometries but is very time-consuming. By combining both methods for the first time, the weaknesses of both methods could be compensated. The phototrophic cyanobacterium *Tolypothrix distorta* was cultured in a moving bed photobioreactor inside a biocarrier with a semi-enclosed geometry. An automated workflow was developed to process µCT scans of the biocarriers. This allowed quantification of biomass volume and biofilm-coverage on the biocarrier, both globally and spatially resolved. At the beginning of the cultivation, a growth limitation was detected in the outer region of the carrier, presumably due to shear stress. In the later phase, light limitations could be found inside the biocarrier. µCT data and biofilm thicknesses measured by OCT displayed good correlation. The latter could therefore be used to rapidly measure the biofilm formation in a process. The methods presented here can help gain a deeper understanding of biofilms inside a process and detect any limitations.

## 1. Introduction

The majority of all known microorganisms in nature are found as biofilms. This means they grow on surfaces in groups of the same or other microorganism species and surround themselves with a highly hydrated matrix of extracellular polymeric substances (EPS) consisting of polysaccharides, fatty acids, enzymes and DNA, among others [1]. This matrix performs various functions; in addition to providing mechanical cohesion for the cells, it serves as a nutrient and water reservoir or as reaction space for extracellular enzymes [2]. There are several good reasons to use microorganisms as biofilms instead of suspension cultures in bioprocess engineering: First, many strains grow only as biofilms and cannot be cultivated in any other way. Second, many microorganisms change their metabolism if growing as biofilm, making some target products economically accessible at all [3]. Third, microorganisms in biofilms are better protected from external stress factors, such as toxins found in wastewater, for example. Biofilms have been used for over 30 years in moving bed biofilm reactors (MBBR) [4]. In this process, plastic biocarriers with a large surface area protected from mechanical stress are used in a fluidized bed reactor, on which microorganisms of sewage sludge form a mixed-species biofilm. Furthermore, immobilization offers the advantage that, in continuous cultures, the dilution rate can be freely selected and no washing out of the cells takes place, independent of the growth rate of the microorganisms [5]. So far, MBBR technology has been used primarily with heterotrophic microorganisms. However, wastewater treatment with microalgae or cyanobacteria is also possible. These phototrophic microorganisms are able to absorb nutrients such as phosphates and nitrates as well as heavy metals from wastewater, without a carbohydrate source [6]. The resulting biomass can be used, for example, as food, fuel, or feedstock in a biorefinery [7,8]. A particular challenge in the cultivation of microalgae is the light supply, which is not dispersible [9]. Especially when cyanobacteria grow as a biofilm, it is important to expose the whole biofilm to sufficient light and avoid shading in some areas of the biocarrier.

A major hurdle for the use of biofilms in process engineering is the quantification of the biomass of biofilms, as well as the investigation of emerging heterogeneity. Determination of the cell dry weight (cdw) is usually only possible destructively and is a very coarse method. The variety of microscopic methods, such as optical [10,11] scanning electron [12,13], atomic force [14,15], and laser scanning microscopies [15,16,17,18,19], often have shallow penetration, especially in optically dense materials. These methods to characterize biofilms also show limitations due to measuring principles or sample preparations. Optical microscopy offers no possibility to investigate biofilms grown on porous media, while scanning electron microscopy needs dehydrated samples [12]. Confocal laser scanning microscopy is limited due to the shallow penetration of the sample [12]. For this reason, more tomographic methods are being developed, such as well-established optical coherence tomography (OCT) [20,21,22,23]. Here, the measuring principle is based in the light interference of sample material to a reference material. Measurement of inhomogeneous materials leads to different indices of refraction and combined with the reference signals, an interference pattern is established, which can be recorded by a detector [24]. The OCT allows for measurement of a biofilm non-invasively and almost in real time; however, it is suitable only for easily accessible positions or samples when good light penetration is guaranteed. 

In contrast, X-ray microtomography (μCT) allows for measurement of biofilms and structures non-invasively, inside and outside, for example, complex flow channels [25], glass capillaries [17], central venous catheters [26], or porous media like 3-D-packed bead columns [27]. The measurement of µCT is based on the x-ray attenuation of matter. Here, the x-rays pass the sample and are recorded on a detector. There, the projection images of the samples are created. Afterwards, an algorithm can be used to transform the x-ray projection images into cross-sectional images by a reconstruction. With these reconstructed images, the image analysis can be performed. This gives access to a broad spectrum of applications in research, for example, studying of pore network geometry, flow and mass transfer [28], modeling of pore scaling and upscaling [29], or even biofilm distribution on porous media [30]. The µCT technique is not only a useful tool to visualize bacterial communities, it also allows the possibility of insight into the structures of bacterial biofilms and the relationship between biofilm structure and its functions, for example, biogenic calcite minerals as diffusion barriers [31]. Often µCT scans are used to obtain a 3D model for a simulation (e.g., CFD) or for 3D printing [32]. Due to poor contrast differences in μCT analysis between biofilm and biocarrier [33], often a contrast agent is needed. Until now, a broad variety of contrast agents have been studied, but no universal contrast agent has been found [34]. 

In this study, we investigated the formation of a phototrophic biofilm of the cyanobacterium *Tolypothrix distorta* on a high-density polyethylene (HDPE) biocarrier in a photobioreactor as a model system. Several biocarriers were taken regularly from the reactor as samples and the biofilm was measured by OCT and μCT. Using μCT, complete scans of the biocarrier could be acquired; this allowed us to determine both the total volume and the degree of surface coverage with biomass by the use of our newly developed workflow. In addition, we were able to quantify the thickness of biofilm through spatial resolution. Due to the rather similar attenuation of biomass and carrier, a contrast agent, in this case Lugol’s iodine solution, was used to enhance the contrast, thus allowing the correct characterization of the biofilm. OCT was used to determine biofilm thickness. Both methods were also compared with cell dry weight. 

## 2. Materials and Methods

### 2.1. Cultivations and Reactor

**Figure 2:**
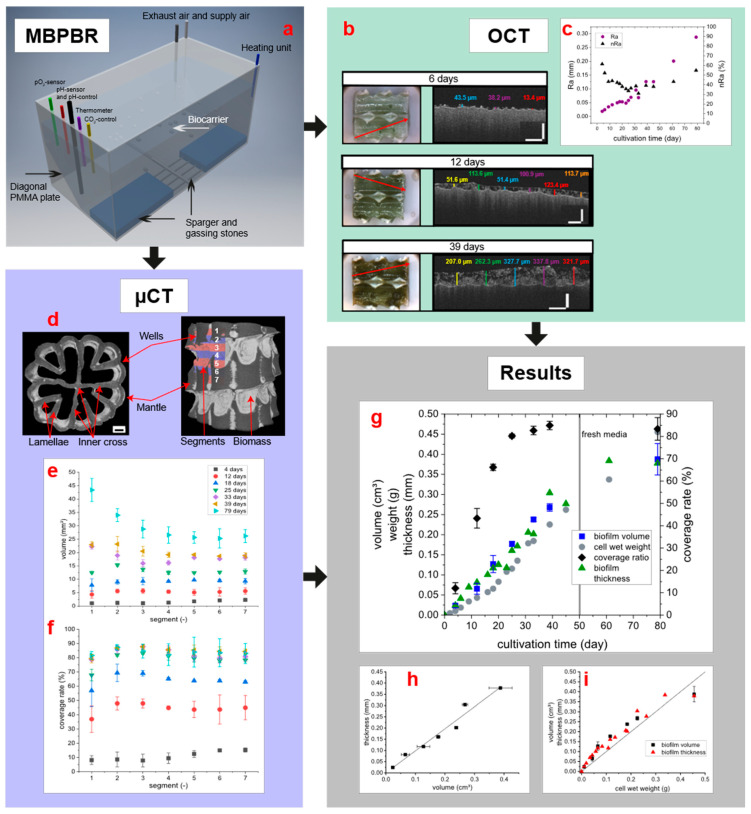
Monitoring the development of the biofilm of *T. distorta* using μCT and OCT. (**a**) Illustration of the moving bed photobioreactor (MBPBR) used for phototrophic cultivation of *Tolypothrix distorta* on the biocarriers. (**b**) Representative optical coherence tomography (OCT) 2-D scans of the carriers at different cultivation times. Left: Photographs of the prepared carriers (cut open), the scan position is indicated in red. Right: Sections of the OCT scans, with the measured thickness of the biofilm plotted. The carrier is located below the biofilm. Scale bar corresponds to 250 μm. (**c**) Arithmetic roughness and normalized (to respective mean) arithmetic roughness of the biofilm over the cultivation period (*n* = 1). (**d**) Overgrown biocarrier (left: 4 days of cultivation, right: 79 days) with marked important areas and segments shown in red and blue, respectively. Scale bar corresponds to 1 mm. (**e**) Distribution of biomass volume on different segments over cultivation time (mean and standard deviation, *n* = 3). (**f**) Surface coverage of biomass, divided in different segments, over cultivation time (mean and standard deviation, *n* = 3). (**g**) Development of biofilm on biocarriers during the cultivation in MBPBR. After 50 days, the entire supernatant, including suspended cells, was removed and replenished with fresh media. Volume and surface coverage of biofilm were measured by μCT (mean and standard deviation, *n* = 3); median biofilm thickness was measured by OCT (*n* = 164–204). Volume and weight are provided for a single biocarrier. (**h**) Correlation between the biofilm volume or thickness to biomass over the entire cultivation period. The straight line is a bisector. **i:** Correlation of median biofilm thickness and biofilm volume. The line is a linear function, with slope 0.98 ± 0.04 and R^2^ of 0.98. To calculate all cww values, the measured cdw values were divided by the correlation factor 0.0673.

The model cyanobacteria strain (*Tolypothrix distorta var. penicillata* *BB97.19)* was obtained from the former strain collection of Professor Büdel of the Department of Plant Ecology and Systematics, TUK. The strain was chosen because it releases antibacterial active substances into the supernatant, and therefore should be cultivated on a larger scale. For strain maintenance, it was cultivated under phototrophic conditions at 17 °C and an illumination intensity of 20 μmol m^−2^ s^−^^1^ in Z-8 media [35]. *T. distorta* was expanded over several scales (500 mL shaking flask (no baffles, 4 weeks, 24 °C, 100 rpm, and 100 μmol_photons_ m^−2^ s^−1^), 5000 mL shaking flask (no baffles, 4 weeks, 24 °C, 100 rpm, and 100 μmol_photons_ m^−2^ s^−1^), 10 L stirred tank (no baffles, three inclined blade impeller, 50 rpm, 4 weeks, room temperature, external illumination with four 24 watt fluorescent tubes)). Then biomass (18.54 g cell wet weight (cww) respective 1.31 g cell dry weight (cdw)) was transferred to a self-built 65 L moving-bed-photobioreactor (MBPBR). In addition, 1585 g of autoclaved HXF 14 KLL biocarriers (Hel-X Biocarriers; Christian Stöhr GmbH& Co. KG, Marktrodach, Germany) (equals ~3100 pieces) were added to the reactor. These biocarriers have a cylindrical shape with a diameter and height of 14 mm (see Figure 2d). The manufacturer specifies the total surface per biocarrier as 25.8 cm^2^ and the protected surface (total, without outside mantle) as 21.7 cm^2^. Carrier material was white high-density polyethylene with, according to our own measurements, a material density of 995.00 ± 0.91 kg m^−3^ and a contact angle for water in air of 92.28 ± 4.29°. The reactor was made of glass, had a rectangular footprint (80 cm × 40 cm × 35 cm) and a ramp formed by a glued-in PMMA plate (Figure 2a). The MBPBR was aerated by gassing stones with 10 L min^−1^ of pressurized air. The gassing kept the biocarriers in suspension, and the ramp supported this by inducing a circulating flow. An illumination was provided externally in a 16:8 daily rhythm (averaging 206 μmol_photons_ m^−2^ s^−1^ on the surface of both long sides, with warm white (2700 K) and cold white (6300 K) (ratio 1:1) LEDs). The reactor system will be described and characterized in detail elsewhere (manuscript in press). The pH was not regulated, and no additional CO_2_ was supplemented. Batch cultivation was performed in the reactor for 45 days, with a sample taken every 2–3 days. Thereafter, the medium was completely replaced, and two samples were taken after 61 and 79 days. Evaporated water was regularly refilled with deionized water. 

### 2.2. Biomass

A distinction was made between suspended cdw and immobilized cdw on biocarriers. In order to determine the suspended cdw, a sample of 100 mL was taken from the reactor. The biomass was separated from the medium by centrifugation (3000 g, 10 min), dried at 80 °C for 24 h, and then the cdw was determined gravimetrically (*n* = 1). For the immobilized cdw, 10 biocarriers were removed from the reactor, dried at 80 °C for 24 h, and weighed (*n* = 1). The following washing with 50% sulphuric acid (for 1 h at 150 rpm and 25 °C) dissolved the biomass, while the biocarriers were only mildly attacked (0.005 ± 0.002 mg mass loss per biocarrier, which is less than 1% of the lowest measured cdw (0.588 mg)). Biocarriers were then rinsed with water, dried for 24 h at 80 °C, and weighed afterwards. The mass difference corresponded to the cdw on biocarriers. For a better comparability of biomass with biofilm volume, a cell-wet-weight (cww)- to cdw-correlation was established at one time-point. Therefore, excess water was absorbed from biocarriers with paper towels and the complete wet biomass of 3, 6, and 9 biocarriers was mechanically scrapped of, collected, weighed, and dried for 24 h at 80 °C. Dried biomass was measured and a correlation coefficient of cww to cdw was determined. For better demonstration, the measured cdw values were converted to cww values with this correlation because the correlation was only created at one point; the actual cdw values are more precise, which can be calculated by multiplication with the factor 0.0673.

### 2.3. OCT

A spectral domain OCT (sdOCT; Thorlabs, Newton, NJ, USA) was used to determine the thickness of biofilm. Half of the mantle, including the lamellae of a biocarrier, was carefully removed using a plier without damaging biofilm in the area of interest. Then, the biofilm on the inner cross areas (approx. 14 mm × 7 mm) was measured in air (see Figure 2b). Every 2–3 days, 5 biocarriers were measured and two 2-D scans were performed per biocarrier. In each 2-D scan, thickness of the biofilm on substrate was measured at measuring points that were each 0.5 mm apart (*n* = 164–204).

In addition to determining the thickness of the biofilm, OCT data was also used to calculate the arithmetic average roughness *(Ra)* according to Tang [36]:

(1) $$Ra=\frac{1}{N}\sum_{i=1}^{N}\left|Xi\right|$$

with *Xi* as the thickness (mm) of the biofilm at point *i* and *N* as the quantity of points measured on that day (*n* = 1). In addition, a normalized roughness (%) was calculated:

(2) $$nRa=\frac{Ra}{mean}*100$$

where *mean* is the mean value of the biofilm thickness measured on that day (*n* = 1).

For the µCT measurements, Lugol’s solution was used as the contrast agent, which has the potential to change the morphology of the biofilm. To investigate this, the biocarrier mantle was removed and the biocarrier was fixed firmly in a small bowl under OCT measuring head. Lugol’s iodine solution was added until the area under investigation was just covered with solution. Next, OCT was focused, and measurement started. 2-D scans were taken at regular time intervals on the inner cross. A total of six carriers were measured, each over 10 min with 30 s measurement intervals (*n* = 6). In addition, three carriers were measured, each over 5.5 h with 30 min measurement intervals (*n* = 3). In each 2-D scan series, the thickness of the biofilm was measured at exactly the same spot on the x-axis at a distance of around 0.5 mm.

### 2.4. μCT

#### 2.4.1. Sample Preparation

Samples were taken each 6–8 days before media exchange; afterwards, before the end of cultivation (79 days), a sample was collected for μCT measurement (3 replicates measured). The samples were stored in a fridge (5 °C and without light) before μCT measurement, while sample preparation and µCT measurement were done at room temperature. A single biocarrier was taken and the remaining cultivation supernatant was removed by draining. The biocarrier was then treated with contrast agent Lugol’s iodine solution (stock solution: first 100 g L^−1^ potassium iodide then 50 g L^−1^ iodine crystals dissolved in deionized water; before measurement, the stock solution was diluted 1:4 with deionized water) for 10 min at room temperature. Excess contrast agent not absorbed was completely removed by using paper towels. Modeling clay was used for fixation of the biocarrier in a sample container (12 mL LDPE sample vial; Nalgene, ThermoScientific, Rochester, NY, USA) and then the sample container was placed in the μCT.

#### 2.4.2. Scan and Scan Settings

Scans were performed using an X-ray microtomograph (Skyscan 1272, Bruker microCT N.V., Kontich, Belgium). Scanning settings were 60 kV, 160 μA, and 0.3° angular steps, with a resolution of 6.5 μm pixel size. To enhance scan quality, 360° scans were performed with frame averaging of four images and random movement of 10 pixels.

#### 2.4.3. Reconstruction

For reconstruction of the μCT image stacks, the software NRecon (Version 1.7.4.6, Bruker microCT N.V.; Kontich, Belgium) and reconstruction engine InstaRecon^®^ CBR Server Premium™ 15K (Version 2.0.2.6, InstaRecon Inc.; Champaign, IL, USA) were used. Parameters for reconstruction were smoothing 4, ring artifact reduction 20, and beam hardening reduction 55%.

### 2.5. Image Analysis

The reconstructed images were analyzed with the software tool CT Analyser (Version 1.19.11.1, Bruker microCT N.V.; Kontich, Belgium). For this purpose, a task list using various plug-ins for image analysis was compiled. By using this task list, the entire biocarrier and the attached biomass could be analyzed in one workflow.

**Figure 1:**
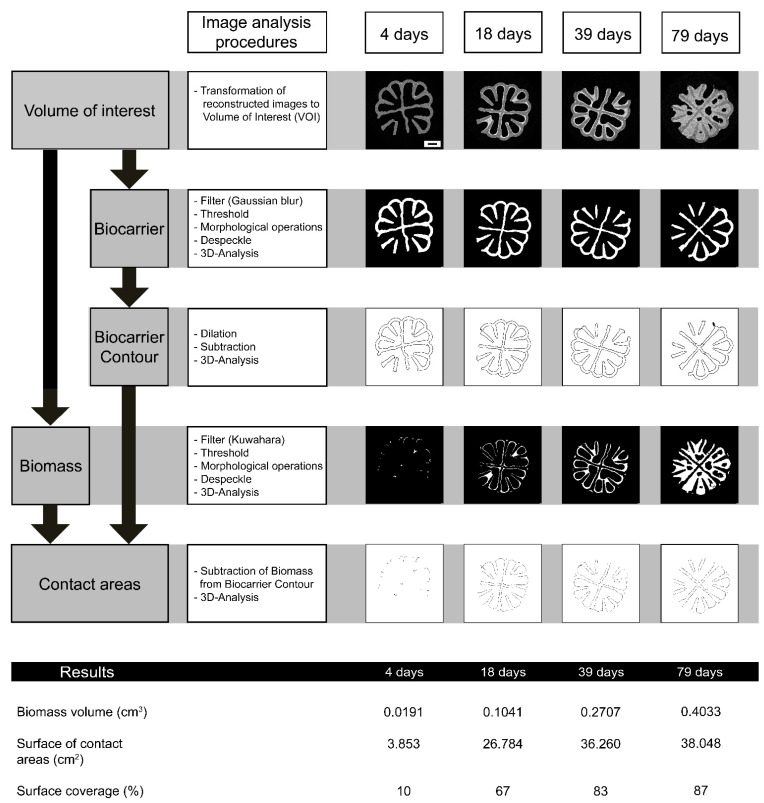
The steps of image analysis and examples of X-ray microtomography (µCT). The procedure of image analysis (left part of graphic), where the volume of interest (VOI) is used to determine biocarrier and biomass. The biocarrier can be used to determine its contour. Contact areas can be determined by a combination of biocarrier contour and biomass, as shown in the last lane of white images in this graphic. Here, only one replicate per cultivation is shown with its original image analysis results by one image of the stack. The results were shown for different cultivation times: early cultivation (4 days + 18 days), middle time (39 days), and late times of cultivation (79 days). Note: The images of biocarrier contour and contact areas were inverted due to better visibility. Finally, some resulting numbers are shown beneath the contact areas. Here, increasing biomass volume and surface coverage with rising cultivation time are also shown. Scale bar (on VOI image of 4 days) corresponds to 2 mm.

First, a volume of interest (VOI) was defined that encloses the biocarrier; second, the wall of the sample container was excluded. Finally, image analysis (see Figure 1) can be started, according to the analyzing task list. Table S1 shows the detailed workflow with the exact settings of the CTAnalyser.

The focus of image analysis was set on the following points: Biocarrier, Biocarrier contour, and Biomass and Contact area of biomass with biocarrier. Figure 1 shows these major parts of the image analysis on the left side as grey boxes and the order of the analyzing process directly next to it. In these white boxes the individual plug-ins are listed, which are necessary to achieve the results of the individual analysis steps on the basis of the VOI images. Some examples of these analyses are shown on the right side and will be discussed in Section 3.4.

For the analysis of biocarriers and biomass, filters (Gaussian blur and Kuwahara, respectively) were used before thresholding. These filters improved binarization results and facilitated further processing of the images. Due to artifacts or measurement noise, some isolated pixels remained after thresholding, which required a plug-in to remove speckles. Morphological operations such as Erosion, Dilation, and Closing were used to model and adjust the analysis target to improve congruence of the object and the original image. To determine the biomass, the original images need to be reloaded and processed with a suitable filter and adjusted threshold. Once biomass has been analyzed, determination of contact areas can be done using the previously stored biocarrier contour results.

Bitwise operations are now used to work out the contact areas of the biocarrier and biomass. Exact parameters and detailed workflow can be found in Table S1. The central element of this analysis is the carrier contour, which was worked out in the second image analysis step. The biomass is now subtracted from it. The result is a structure that includes all areas where the biomass and carrier contour are not in contact with each other. However, the targets of the analysis are the contact areas of the biomass with the biocarrier. For this reason, the determined structure without contact areas is subtracted from the carrier contour. This results in a structure that includes all contact areas of the biomass with the biocarrier.

Finally, a 3D analysis was performed to determine parameters such as object volume or object surface. This can be done by creating a 3D surface-rendered volume of the object within the VOI. The marching cubes method [37] is used for rendering. Calculation of morphometric parameters such as object volume or surfaces happen according to this 3D model. Based on the surface data of biocarrier contour and surface, the contact areas with biomass was determined as follows:

(3) $$Surface\;coverage\;\left[\%\right]=\frac{surface\;of\;contact\;areas\;of\;biocarrier\;and\;biomass}{surface\;of\;biocarrier\;contour}*100$$

To investigate how much the biofilm coverage varies across the biocarrier surface, segments were defined (in Figure 2d, highlighted in red and blue excerpts). Since not every biocarrier had the same spatial orientation in the sample container, the position of segments was determined on the basis of the center cross. To keep analysis comparable, it was decided that the first zone starts as soon as the inner cross is fully visible on reconstruction images. One segment consists of 154 images (corresponding to 1 mm biocarrier height), and a total of 7 segments (about half of the carrier) were analyzed according to the methodology described above. However, due to the symmetry of the biocarrier, the results can be assumed for the whole structure. Analyzing task lists were adapted to the small size of the segments and parameters, for example, Despeckle operations were adjusted; general procedures remained the same. Exact values and parameters of all image analysis processes in CT Analyser can be found in Table S1 for an entire biocarrier and Table S2 for analysis of the biocarrier segments.

## 3. Results

The aim of this study was to monitor the development of biofilm on the biocarrier over the cultivation period. For this purpose, the already established OCT was combined with a new self-developed method for µCT, which has rarely been used. To visualize the interaction of these methods, all important results are presented in Figure 2. Here, Figure 2a shows the reactor from which the biocarriers were taken for the different analysis methods, while Figure 2b and c show representative images of the OCT and the roughness of the biofilm determined by OCT. Figure 2d demonstrates two exemplary 3D models of overgrown biocarriers. Below these biocarriers, Figure 2e,f represent the results of the spatially resolved µCT analyses for biomass volume and coverage rate. The bottom right presents the results obtained by combining the methods; Figure 2g presents the progression of biofilm on the biocarriers over the cultivation period; while Figure 2h,i show the correlations between the methods. In this chapter, the results are presented sorted by method. The subsequent discussion chapter then addresses their combination.

### 3.1. Biomass Monitoring

The development of *T. distorta* biomass in the reactor, either as a planktonic or biofilm, during batch operation was determined (Figure S1). Already, in the first sample on the second day, biomass was measurably immobilized on biocarriers (0.014 g L^−1^ immobilized cdw). In the first growth period, both the planktonic biomass and the biofilm grew with high growth rates (day 2–6 μ_suspension_ = 0.90 day^−1^ and μ_biofilm_ = 0.34 day^−1^). Subsequently, the growth rate decreased due to several limitations until day 15. Fine biomass in the supernatant caused shading and thus partial light limitation in the reactor. In addition, both sulfate (after day 15) and phosphate (after day 20) were depleted in the supernatant. Nitrate was not completely depleted throughout the batch cultivation period (180 mg L^−1^ after 18 days, 16 mg L^−1^ after 45 days; measured by Ion-Chromatography, see Figure S2); as a result, the concentration of biomass in the suspension decreased. In contrast, the immobilized biomass continued to increase, with a reduced growth rate of 0.07 day^−1^ between days 9 and 33, after which it decreased to a slightly lower value. The growth rate of total biomass decreased from 0.50 day^−1^ to 0.04 day^−1^ during this period.

### 3.2. Biofilm Monitoring Using OCT

Due to physical properties, the OCT cannot measure through the HDPE material of the biocarrier into its inside. However, most of the biofilm grew inside it, so the biocarrier had to be prepared before OCT measurement. For this purpose, the mantle including lamellae was carefully mechanically removed without damaging the biofilm, before measuring. The Biofilm on the mantle and lamellae could not be measured because it would have been destroyed during preparation. Therefore, OCT could only measure the biofilm on the middle cross (compare Figure 2d) over the cultivation period (see Figure 2b). The thickness of biofilm increased linearly in the first 40 days at a rate of 0.0065 mm day^−1^ (R^2^ 0.96) to a thickness of 0.29 mm (Figure 2g). Maximum thickness was achieved at 0.38 mm. Biofilm formed by *T. distorta* was very heterogeneous. Roughness was calculated according to Tang et al. [32] by the use of Equations (1) and (2) and is shown in Figure 2c. The roughness exceeded 50% of biofilm thickness during the first 10 days and stabilized to 40% after. On day 79, however, the roughness increased again to 55%.

### 3.3. Effect of Lugol’s Iodine Solution on the Biofilm

Prior to μCT scanning, a biocarrier was immersed in Lugol’s iodine solution for 10 min. Short-term (10 min) and long-term effects (several hours) of this contrast agent on the biofilm were observed by OCT. In the short-term measurement, a statistically significant increase in biofilm thickness of 2.1% (two tailed t-test, significance level 0.05, and degree of freedom 29 (a total of 5 biocarriers with overall 29 measurement points)) was observed after 10 min. The examined biofilm had an average thickness of 0.292 ± 0.213 mm. For quantitative evaluation of μCT scans, a correction factor of 2.1% was introduced to compensate for this swelling effect during the first 10 min. A steady increase was observed over the course of long-term measurement (see Figure S3). A statistically significant increase occurred only after 2 h with a 3.5% increase in biofilm thickness (two tailed t-test, significance level 0.05, and degree of freedom 25). In 4 h, the biofilm swelled by 8.3% and stagnated for the remaining 1.5 h of this experiment.

### 3.4. Biofilm Growth Measured by μCT

The development of the biomass volume, measured by μCT, demonstrated a linear progression until the 39th day of cultivation, with a volume increase of 7.3 mm^3^ (R^2^ 0.98) per day and biocarrier, as presented in Figure 2g. After this cultivation period, the growth of *T. distorta* was slower and the biomass volume increased about 3 mm^3^ per day per biocarrier (R^2^ 0.91). In general, the results of biofilm growth monitored by μCT can be divided into three time periods: early (4–18 days), middle (25–39 days), and late (>39 days to end at 79 days) cultivation time. At the beginning (day 4), only a small amount of biomass adhered to the biocarrier, mainly on protected areas such as wells and small spaces between the lamellae. This can be seen in the biomass and contact area images for 4 days in Figure 1, and also in the image of the overgrown biocarrier 3D model in Figure 2d left, where the bright grey parts are adhered cyanobacteria.In general, the biofilm grew on the inner side of mantle, the inner cross and on the lamellae. The used MBBR biocarriers are designed for to grow biomass only inside. The outside regularly collides with other biocarriers and the reactor wall, and therefore steadily mechanically abrading any biofilm. At the outside only in the deep wells biofilm adhered as can be seen in Figure 1 on the images of biomass and contact areas for either 4, 18, 39, or 79 days. However, it can be observed that the amount of biofilm that has adhered in the wells is becoming smaller, compared to the total biofilm. This can be found in Figure 1 when the biomass is considered.

By determining the biomass distribution, the contact surfaces of biocarrier contour and biomass can be examined. Figure 1 shows an example of the development of the contact surfaces over the cultivation time. After only 18 days, the surfaces of the biocarrier were already covered by a thin biofilm layer. With increasing cultivation time, the biofilm thickness also increased, but contact areas led to a maximum when all protected surface areas were covered. As a result, the surface coverage reached a maximum at 84% (see Figure 2f,g) and only outliers showed a higher degree of surface coverage.

To analyze the biomass distribution over individual biocarriers, they were divided into seven segments, which cover about half of the biocarrier (Figure 2d, right). Hence, it was possible to resolve the surface coverage and the biomass volume over the biocarrier height, and thus obtain information on biofilm growth as a function of the spatial coordinate on the surface of the biocarrier. At the beginning of cultivation (day 4), the biofilm adhered mainly to the inner segments (Figure 2e,f, segments 5–7) with 15% surface coverage, exceeding other segments with only 10%, respectively. With increasing cultivation time, it was noticeable that in segment 1 the surface of biocarrier was less covered with biofilm compared to the other segments. This trend continued during cultivation, as displayed in Figure 2f. However, while curves from 12 to 25 cultivation days showed the trend of highest surface coverage in segments 2 and 3, differences between segments 2 to 7 decreased in the middle and long cultivation periods. A closer look at biomass volume in each segment (Figure 2e) confirmed the same trends as in surface coverage. For example, biomass volume after 4 days was also higher in segments 5 to 7 (with amounts of 1.69 mm^3^, 2.07 mm^3^, and 2.23 mm^3^, respectively), while the other segments had less biomass volume (mean of 1.1 mm^3^). Furthermore, a slightly lower biomass volume was observed on segment 1 at the beginning to middle cultivation period (12–25 days). In contrast, this trend changed after 33 days of cultivation, and, thus, segment 1 had the highest biomass volume. This can be observed clearly in the curve of 79 days, where a biomass volume of 42.5 mm^3^ was determined on segment 1, compared to 33.3 mm^3^ on segment 2 or 28.1 mm^3^ on segment 3. Only from segment 4 onwards, biocarrier volume remained almost the same at the end of cultivation.

## 4. Discussion

One of the most established biofilm processes is the MBBR technology. The biocarrier-type used in this study is applied in a wide variety of different MBBR processes. Due to its semi-enclosed geometry, measuring the development of biofilm over time is challenging. To overcome these obstacles, three different methods were combined: Cdw determination, μCT and OCT. The μCT allows spatial resolve of the biofilm on the entire biocarrier. OCT was used as a reference method because it can measure living biofilms very quickly, easily, and inexpensively, and is therefore one of the best-established methods for this purpose. Measurement and evaluation of the data is very fast, making the method suitable for online monitoring of a process. With OCT, only biofilm on the inner cross could be measured, but other areas could not be mechanically prepared without disrupting the biofilm. The determination of cdw was used as a reference to validate the results of the other methods.

The biofilm aged during cultivation, which means besides the increase in biomass and volume, its morphology (e.g., roughness) and composition (density) changed. The absolute roughness (Ra) increased steadily with increasing biofilm thickness over the course of cultivation. Mueller et al. [38] studied phototrophic biofilms with a CLSM in a flow cell and found that roughness first increased over time and then approached a steady state. In contrast, Tang et al. [36] observed three phases, first an increase, then a reduction, and afterwards a long stationary phase. However, an exactly opposite pattern has also been observed [39]. Thus, the absolute roughness as well as the change in roughness over time are predominantly organism-specific. Nevertheless, for almost all biofilms, it can be assumed that a higher shear rate produces a smoother and more compact biofilm [40]. Even though the flow in our reactor remained constant, it can be assumed that it changed within the biocarriers. As the biofilm occupies more and more volume in the biocarrier over time, the free flow cross-section decreases. This reduces the velocity of the flow in the biocarrier [41], which may account in part for the increase in roughness.

A lower flow can also result in a decrease in the density of biofilm, but the opposite was the case. As shown (Figure 2i), the biofilm thickness measured by OCT for the entire biocarrier correlated very well linearly with biomass volume measured by μCT over the entire cultivation period. The growth on the inner cross therefore proved to be representative for the whole biocarrier, so that OCT can be used for routine measurements of the biofilm in this case. The cww consisted of about 93% water (calculated by cdw-cww correlation), so a density of the cww of about 1000 kg/m^3^ can be assumed. Thus, 1 g cww should correspond to a biofilm volume of about 1 cm^3^, as also can be derived from Figure 2h. However, the relationship between volume and mass (density) is not linear over the entire period; density increased towards the end of cultivation. As we already observed in another system with a different cyanobacterium, the density of the biofilm increased over time [23]. The ratio of cells to EPS in the biofilm is likely responsible for this. As we have shown before, the proportion of EPS changes during the cultivation of cyanobacteria [42,43]. Since EPS has a very high water content [2,44], the ratio of volume to cdw changes during cultivation.

To keep the energy, which is required to suspend the biocarriers, low, in most fluidized bed processes biocarriers have a density close to water. Since the biomass consists largely of water, the resulting biofilm density is also close to water. As a consequence, μCT was initially unable to distinguish between biocarrier and biomass so a contrast agent had to be established. After preliminary tests, Lugol’s iodine solution was selected because it sufficiently enhanced the contrast due to the rapid diffusion of potassium iodide into biofilm [27]. However, treatment with a highly concentrated salt solution can potentially lead to a change in the biofilm (e.g., swelling or dehydration), which is difficult to predict. For example, diatoms, dinoflagellates [45], as well as cyanobacteria [46] were found to behave differently by treatment with Lugol’s iodine solution, depending on the species. For the relevant test period (10 min) during which the biofilm was in contact with the solution, only a relatively small swelling of biofilm by 2.1% was detected. This was considered by a correction factor of −2.1% for the biomass volume in μCT results. Due to expected long-term damage to the cells by the contrast agent, biomass cannot be used further [33]. Using a contrast agent can also lead to artifacts which could appear in the form of band-like, linear shaped areas or pixels that were too bright. Some of these effects could be reduced by reconstruction, therefore problems in image analysis caused by artifacts were minimized and, in some cases, could be completely removed with Despeckle plug-ins by the software CT Analyser. Overall, the artifacts in µCT were fewer and only present in a small amount of scan data; so, the impact on image analysis results is small.

With μCT, it was possible to analyze the biofilm distribution. For reasons of image analysis, this was only carried out quantitatively along the axis over segments (e + f). Radially, only a qualitative evaluation can be made (see Figure 1). In the initial biofilm formation phase (day 4), axial distribution demonstrated that biomass grew preferentially in the center of the biocarrier. As shown by CFD simulation elsewhere [41], in a cylindrical biocarrier in a MBBR, the lowest flow velocity prevails in the center of the biocarrier. It is known that lower shear rates promote biofilm formation of cyanobacteria [47]. The inner segments 6 and 7 appear to offer the biofilm a flow protected surface to adhere in early cultivation times. Later (day 39), the axial distribution reversed and there was more biofilm in outer regions (segments 1–3). This indicates a blocking of the biocarrier when biofilm in outer areas adsorbed most of the light and nutrients. Biofilm in the inner segments near the biocarrier core only received light and nutrients through holes in the mantle. The radial distribution in the beginning (day 4) demonstrated an increased formation of biofilm at the wells in the outer part of the mantle and in the inner part of the mantle, at the attachment points of the lamellae and the inner cross. Here, the biofilm is protected from two sides and double wall friction likely reduces flow in this region.

By distinguishing between biocarrier and biomass, the contact areas between these two could be determined. Therefore, first the biocarrier contour was modeled by image analysis and second biomass. Afterwards the contact areas were determined and surface coverage of biocarrier (see Equation (3)) could be calculated with temporal and local resolution. The determined maximum coverage of up to 87% is higher than the percentage of protected surface according to manufacturer’s specifications (84%). This can likely be explained by growth of biomass in the wells, an area which is not considered as protected area. Measured surface of the biocarriers was about 21.93 ± 1.12 cm^2^ per biocarrier (manufacturer’s specification about 25.8 cm^2^). Exact calculations by the manufacturer were not available; therefore, the above data were determined from the CT results. For calculations and 3D-analysis with CTAn, solid objects are generated using the marching cubes method [37], which is an algorithm for rendering 3D models. It should be noted that for biocarriers and biomass, the calculated surface means the outer surface at the object boundaries, while the surface determination at carrier contour or contact areas includes the inner and outer surface of these structures (due to the analysis method). Therefore, the data table in Figure 1 demonstrates a larger surface of contact areas than the determined 21.93 ± 1.12 cm^2^ surface of the biocarrier.

In the μCT scans, it was observed that the biocarrier surface displayed a high surface roughness (exemplarily image can be found in Figure S4). This roughness, especially on inner cross or lamellae structures, resulted in a higher surface of the whole biocarrier. Also, this roughness facilitates adhesion of the biofilm to the biocarrier surface, which is important in early cultivation times, and was observed after 4 to 18 days (see Figure 1) when the contact areas of biofilm increased.

The sensitivity of the two tomography methods used here was different. With OCT, a very thin biofilm was already detectable on almost the entire area examined during the first measurement (day 4). Only about 10% of the measurement points displayed no biofilm at all. At that time, only 10% coverage was determined with the µCT. In contrast, μCT is better for determining the biofilm volume at the middle or long cultivation time, when the biofilm thickness increases. At this point, it becomes difficult to measure biofilm with OCT due to the heterogeneities [23]. Thus, both methods have their strengths and weaknesses, but with a good combination, the growth of biofilms can be monitored well, as shown by the presented data.

## 5. Conclusions

OCT has so far been used very intensively in biofilm research, as it enables fast and cost-effective measurements and data evaluation. However, these examinations usually take place in specially designed systems, such as flow cells, and rarely in technical and scalable systems. Therefore, a biofilm was investigated in a technically relevant MBBR biocarrier. In this biocarrier, measurements with OCT were only possible to a very limited extent. We therefore used the μCT as a supplement, which could measure biofilms spatially resolved and independently of the biocarrier geometry and material (e.g., plastic or minerals) as long as they fit into the device. We were able to show how and where the biofilm builds up based on the biofilm volume and the degree of surface coverage. Thus, at the beginning of the cultivation, the zones of lowest shear stress showed the best growth. However, towards the end, growth was restricted in the core of the biocarrier due to the limited penetration depth of light. Currently, the major disadvantage of μCT is the low speed of measurement. One scan took about 2.5 h, and the complete image analysis currently requires at least 24 h of solely dedicated computing time per scan. The latter could possibly be reduced by hardware and software optimization. Online monitoring of a technical process by μCT alone is thus hardly possible in the near future. However, our data demonstrated that it may be possible to create process-oriented monitoring by cleverly choosing the measuring range of the OCT. Through correlations with μCT and the cdw, it was validated for our system that the measurements of OCT were representative for the whole biofilm. Such setups must be performed again for new systems. Central topics here are the selection and influence of contrast agents as well as the selection of the OCT measurement region. Possible applications of the methods presented here can be the optimization of biocarrier designs. Or, by using the µCT, a representative area in a biocarrier or other surface can be determined beforehand, which can then be monitored online in the process using OCT.

## Data Availability

The dataset used and/or analyzed during the current study are available from the corresponding author on reasonable request. R. Ulber should be addressed for main correspondence. U. Bröckel can be addressed for µCT data materials.

## Supplementary Materials

The following are available online at https://www.mdpi.com/article/10.3390/microorganisms9081743/s1: Figure S1: Distribution of cell dry weight while cultivation, Figure S2: Nutrient concentrations while cultivation; Figure S3: Biofilm thickness after staining with contrast agent, Figure S4: Roughness of biocarriers, and Table S1: Tasklists_CT_Image_Analysis.
