# Supplementary material for: Insights into the Development of Phototrophic Biofilms in a Bioreactor by a Combination of X-ray Microtomography and Optical Coherence Tomography
Source: Microorganisms. 2021 Aug 16;9(8):1743. doi: 10.3390/microorganisms9081743 (PMC8398007; doi:10.3390/microorganisms9081743)
Supplement: Supplementary file 1 [file microorganisms-09-01743-s001.zip › microorganisms-1309382-supplementary/Supplementary Figures.pdf]

2 Supplemental

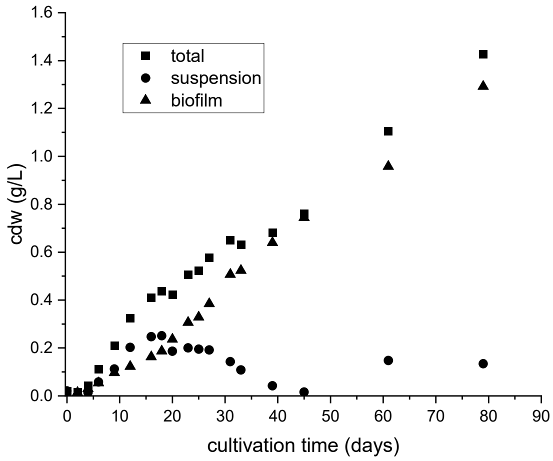

***Figure S1**: **Distribution of cell dry weight while cultivation:** Development of the cdw in the MBPBR. The biomass immobilized on the biocarriers and the suspended biomass were measured. In addition, their sum is presented as total biomass (n=1).*

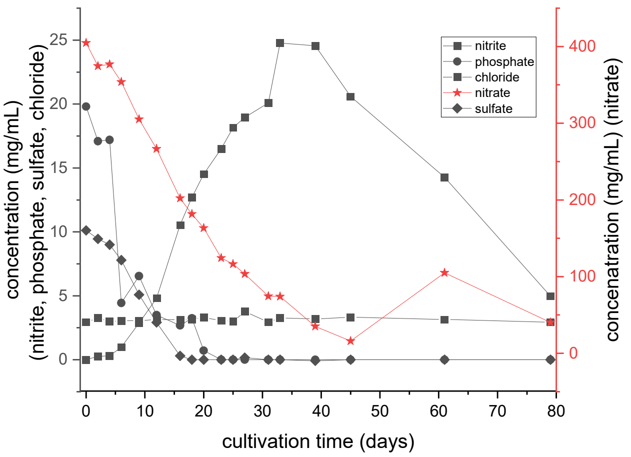

***Figure S2**: **Nutrient concentrations:** Concentrations of various anions in the supernatant in MBPBR during the course of cultivation, measured by ion chromatography. Nitrites, phosphate, sulfates, chloride on the left axis, nitrates on the right axis (n=1). The method was described previously in [42]. The connecting lines are only for better orientation.*

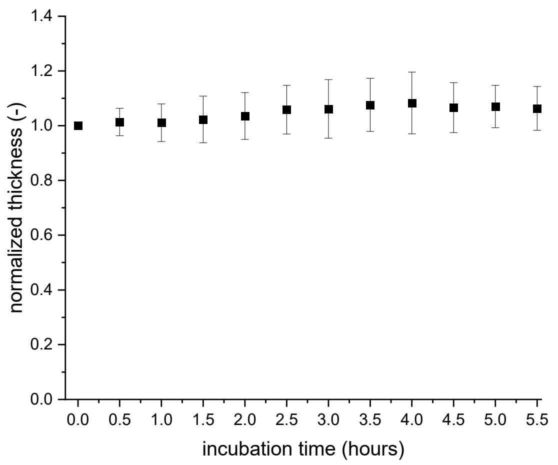

Figure S3: Biofilm thickness after staining with contrast agent: *Evolution of the thickness of the biofilm (measured by OCT) in Lugol's iodine solution (3 carriers, overall 25 measurement points, mean and standard deviation). Mean and standard deviation (n=25).*

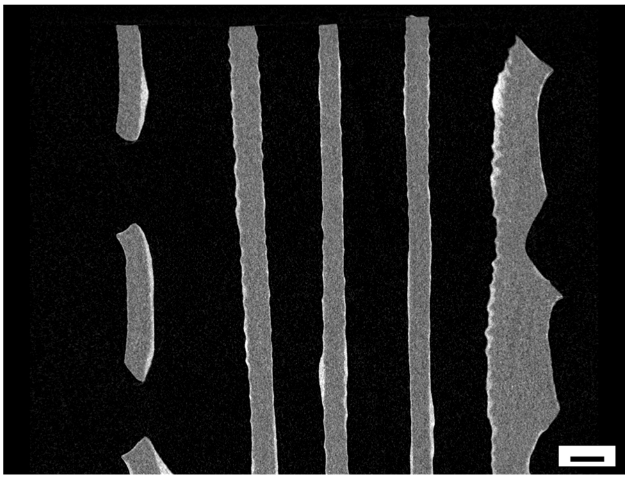

Figure S4: Roughness of biocarriers. *µCT scans enable a sagittal cut through biocarrier (here exemplarily chosen from a scan after 12 days of cultivation). Roughness of biocarrier surface can be seen on either inner cross, lamellae or arches. Scale bar corresponds to 900 µm.*
